# Using a Deep Learning-Based Decision Support System to Predict Emergent Large Vessel Occlusion Using Non-Contrast Computed Tomography

**DOI:** 10.3390/jcm14134635

**Published:** 2025-06-30

**Authors:** Seong-Joon Lee, Dohyun Kim, Dae Han Choi, Yong Su Lim, Gyuha Park, Sumin Jung, Soohwa Song, Ji Man Hong, Dong Hoon Shin, Myeong Jin Kim, Jin Soo Lee

**Affiliations:** 1 Department of Neurology, Ajou University School of Medicine, 164 World Cup-ro, Yeongtong-gu, Suwon-si 16499, Gyeonggi-do, Republic of Korea; editisan@gmail.com (S.-J.L.); dacda@hanmail.net (J.M.H.); 2 Research Division, Heuron Co., Ltd., 10F, C, 150 Yeongdeungpo-ro, Yeongdeungpo-gu, Seoul 07282, Republic of Korea; atown.dk@iheuron.com (D.K.); khp.3927@samsung.com (G.P.); sumini1019@korea.ac.kr (S.J.); soohwa@iheuron.com (S.S.); wadada@iheuron.com (D.H.S.); 3 Department of Neurosurgery, Gachon University College of Medicine, 38-13 Dokjeom-ro 3 Beon-gil, Namdong-gu, Incheon 21565, Republic of Korea; daehan.ns.choi@gmail.com; 4 Department of Emergency Medicine, Gachon University College of Medicine, 38-13 Dokjeom-ro 3 Beon-gil, Namdong-gu, Incheon 21565, Republic of Korea; yongem@gilhospital.com; 5 Department of Neurology, Gachon University College of Medicine, 38-13 Dokjeom-ro 3 Beon-gil, Namdong-gu, Incheon 21565, Republic of Korea

**Keywords:** artificial intelligence, stroke, thrombectomy, reperfusion, neurology

## Abstract

**Background:** This retrospective, multi-reader, blinded, pivotal trial assessed the performance of artificial intelligence (AI)-based clinical decision support system used to improve the clinician detection of emergent large vessel occlusion (ELVO) using brain non-contrast computed tomography (NCCT) images. **Methods:** We enrolled 477 patients, of which 112 had anterior circulation ELVO, and 365 served as controls. First, patients were evaluated by the consensus of four clinicians without AI assistance through the identification of ELVO using NCCT images. After a 2-week washout period, the same investigators performed an AI-assisted evaluation. The primary and secondary endpoints in ELVO prediction between unassisted and assisted readings were sensitivity and specificity and AUROC and individual-level sensitivity and specificity, respectively. The standalone predictive ability of the AI system was also analyzed. **Results:** The assisted evaluations resulted in higher sensitivity and specificity than the unassisted evaluations at 75.9% vs. 92.0% (*p* < 0.01) and 83.0% vs. 92.6% (*p* < 0.01) while also resulting in higher accuracy and AUROC at 81.3% vs. 92.5%, (*p* < 0.01) and 0.87 [95% CI: 0.84–0.90] vs. 0.95 [95% CI: 0.93–0.97] (*p* < 0.01). Furthermore, the AI system improved sensitivity and specificity for three and four readers, respectively, and had a standalone sensitivity of 88.4% (95% CI: 81.0–93.7) and a specificity of 91.2% (95% CI: 87.9–93.9). **Conclusions:** This study shows that an AI-based clinical decision support system can improve the clinical detection of ELVO using NCCT. Moreover, the AI system may facilitate acute stroke reperfusion therapy by assisting physicians in the initial triaging of patients, particularly in thrombectomy-incapable centers.

## 1. Introduction

In the hyperacute treatment of patients with acute ischemic stroke (AIS), the prompt identification of emergent large vessel occlusion (ELVO) and endovascular treatment (EVT) for rapid recanalization are key to improving outcomes [1]. Thus, thrombectomy-capable centers optimize their hyperacute stroke imaging protocols to identify ELVO [2]. However, not all patients attend thrombectomy-capable centers, and many present to primary stroke centers or primary hospitals [3].

The reported median onset-to-revascularization time for ELVO patients in the United States was shorter for direct versus transfer patients, resulting in lower rates of functional independence in the transfer group [4]. Good functional outcomes may rely on shorter transfer times and faster reperfusion in the interhospital transfer system [5,6]. However, there are multiple barriers to this, such as imaging acquisition time, imaging protocol differences across primary centers, and inadequate care from primary physicians or emergency medical technicians. As non-contrast computed tomography (NCCT) is routinely performed in nearly all hospitals for patients suspected of stroke, an artificial intelligence (AI)-based clinical decision support system that can identify ELVO and EVT candidacy based on NCCT images may help streamline interhospital transfers. Furthermore, NCCT may be performed even if the patient only has minor symptoms, even in thrombectomy-capable centers. ELVO identification based on NCCT may also facilitate the streamlining of in-hospital processes.

In this clinical trial, we aimed to validate diagnostic performance improvement with the assistance of an AI-based clinical decision support system that identifies ELVO and EVT candidacy based on NCCT (Heuron ELVO) by clinicians of varying specialties and expertise. In particular, this trial was designed to validate the performance and safety of Heuron ELVO for its approval by the South Korean Ministry of Food and Drug Safety.

## 2. Methods

### 2.1. Study Ethics

The protocols for this clinical trial were approved by the South Korean Ministry of Food and Drug Safety (KFDS-1283, 2021). Data collection and evaluation were approved by the Institutional Review Board of Gachon University Gil Medical Center (GCIRB2022-002). The ethical standards of the 1964 Declaration of Helsinki and its later amendments were implemented, and the need for written informed consent was waived owing to the retrospective data collection method used.

### 2.2. Heuron ELVO for the Identification of Emergent Large Vessel Occlusion

Heuron ELVO is a computer-aided triage and notification software that analyzes NCCT brain images. It is intended to assist hospital networks and trained stroke experts in workflow triage by flagging findings suggestive of ELVO in the internal carotid artery (ICA) or middle cerebral artery (MCA) M1 on NCCT images. To acquire these results, CT image processing and Deep Learning-based triage models are applied.

At least three imaging markers can be identified as indicators of emergent large vessel occlusion on NCCT images. The most representative imaging marker is the hyperdense artery sign (HAS), a phenomenon in which an artery is blocked by a thrombus and appears hyperdense on NCCT images. Although it is the most representative imaging marker of MCA occlusion on NCCT, its presence depends on the slice thickness of the NCCT images. Thus, HAS alone may have low sensitivity when classifying patients in NCCT [7]. A second ELVO imaging marker that can be identified on NCCT is eyeball deviation, which deviates in the direction of the hemisphere of the ELVO. A study that classified patients with ELVO using eyeball deviation observed on CT images as a single indicator reported a sensitivity of 71% and a specificity of 77.5% [8]. A third potential imaging marker is early ischemic change (EIC) following ELVO. EIC represents ischemic changes in brain tissues identified in NCCT images taken at an early stage after stroke onset and is the basis of the Alberta Stroke Program Early CT Score (ASPECTS) [9]. While EIC is not a specific indicator of ELVO, its presence may indicate ELVO.

**Figure 1:**
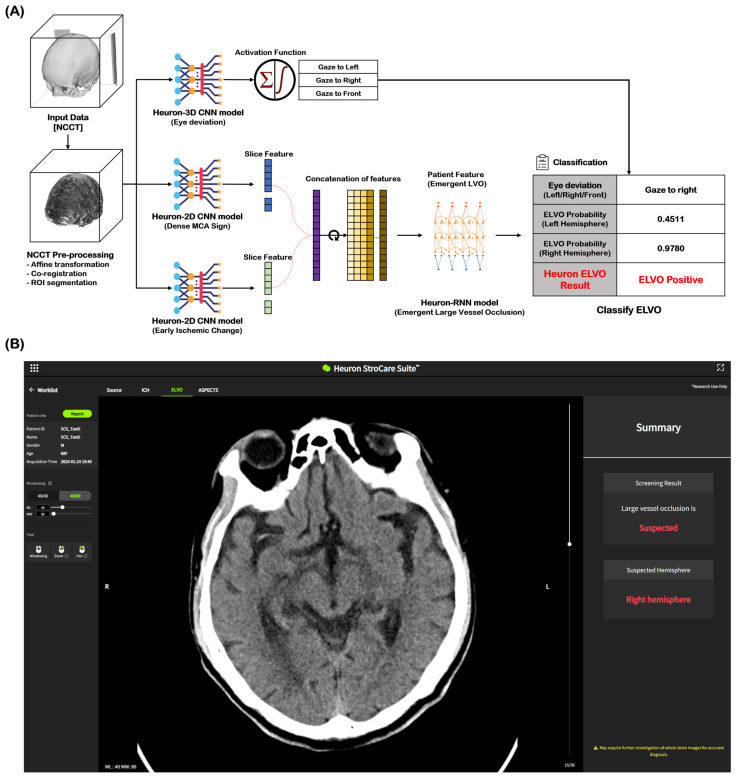
Automatic ELVO classification based on the Deep Learning model. (**A**) Inference flow for classifying patients with emergent large vessel occlusion, and (**B**) an output example of a suspected ELVO case in Heuron ELVO. ELVO, emergent large vessel occlusion; NCCT, non-contrast computed tomography; ROI, region of interest; CNN, convolutional neural network; MCA, middle cerebral artery; RNN, recurrent neural network.

NCCT images of 2184 cases (ELVO: 1202 cases, non-ELVO: 982 cases) were used as the learning model for ELVO classification. The total cases were split in an 8:1:1 ratio for training, validation, and testing, respectively. In both the training and validation datasets for this model, any cases with the same registration ID from the same institution were excluded. Among the imaging biomarkers, HAS and EIC were identified by stroke experts and trained as ELVO imaging biomarkers. Eye deviation was independently identified and included in the learning model by detecting the deviation of the crystalline lens on NCCT. Figure 1A shows the Heuron ELVO process from input to output. After the preprocessed image was delivered to the convolutional neural network (CNN) model, Heuron ELVO identified the presence of eyeball deviation, HAS, EIC, and old infarct (OI). We employed class activation mapping to visualize region-specific feature contributions in NCCT scans, particularly for identifying occlusion patterns. These methods allow us to validate whether model decisions correlated with the radiological features of large vessel occlusions (e.g., hyperdense artery signs or early ischemic change). Subsequently, the features identified using the Heuron-2D CNN models were concatenated and input into the Heuron recurrent neural network model to classify whether ELVO was present, and a probability value between 0 and 1 was calculated. We applied Platt scaling to the validation set for probability calibration. The decision threshold was determined by selecting the point with the highest Youden’s J statistic (J = sensitivity + specificity − 1J = sensitivity + specificity − 1) on the ROC curve (Shown in Figure S1 and Table S1). Figure 1B shows an example of a Heuron ELVO output presented to clinicians.

### 2.3. The Design of the Clinical Trial

This study was a single-center, multi-reader, blinded, retrospective clinical test performed to evaluate the effectiveness of a Deep Learning solution that automatically classifies ELVO patients based on brain NCCT. The primary endpoint was the sensitivity and specificity of ELVO prediction following Heuron ELVO assistance compared to that of unassisted NCCT image analysis only. The sample size for primary endpoint verification was calculated based on the results of previous studies and the results of Heuron ELVO internal testing. In the studies by McCluskey et al. (2019) [8] and Lim et al. (2018) [10], sensitivity and specificity were approximately 69% and 80%, respectively, when clinicians identified ELVO using only NCCT images. In addition, the sensitivity and specificity of the Heuron ELVO in the internal test were 87.1% and 88.8%, respectively. The sample size was calculated with the significance level of the two-tailed test set to 0.05, with 90% power after correction according to multiple tests, and based on the sensitivity and specificity values mentioned above. Accordingly, the number of cases in the ELVO group was 101, and that in the non-ELVO group was 329. Accounting for a dropout rate of 10%, 113 and 366 patients were recruited in the ELVO and non-ELVO groups, respectively.

### 2.4. Study Population and Data Collection

**Figure 2:**
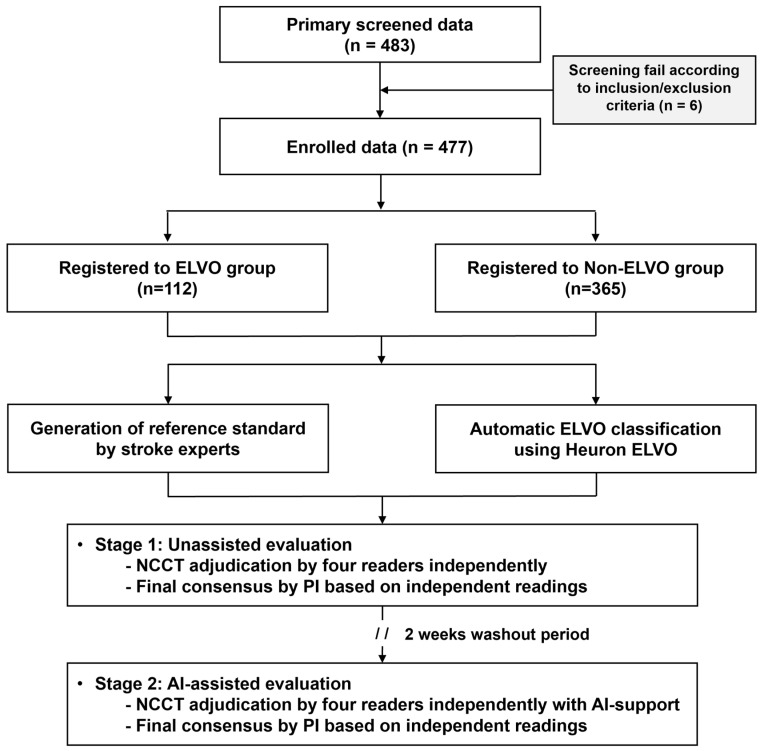
A flowchart of the current study regarding patient selection and clinical trial performance. ELVO, emergent large vessel occlusion.

This trial was conducted at a tertiary hospital (Gachon University Gil Medical Center). When a patient suspected of stroke presents to the emergency department, NCCT is performed first. CT angiography and perfusion imaging are then performed in patients who present within 6 h of symptom onset without hemorrhage and who are likely to benefit from reperfusion therapies. The current study was a retrospective imaging-based clinical trial. Specifically, patients aged ≥19 years presenting to the emergency department between January 2019 and January 2022 and sequentially undergoing NCCT, CT angiography, and perfusion imaging due to suspected acute ischemic stroke were retrospectively grouped based on their ELVO status. The ELVO group included patients in whom ELVO was confirmed using CT angiography, perfusion, and diffusion-weighted MRI, while the non-ELVO group included patients in whom ELVO was not present. Cases were consecutively selected in reverse chronological order for each group until the target number was obtained. During this process, patients were excluded if cerebral infarction occurred elsewhere other than in the anterior circulation, if there was a lack of information, such as metadata or electronic medical records during automatic anonymization or human errors to record, or if there was severe noise in the CT images (Figure 2). All demographic information was anonymized, and an independent ID was provided for this study. In the CT images of registered cases, slight differences were observed in the scanning model of the device, scanning protocol, or parameters, depending on the scan date. However, all were obtained using devices from the same vendor (Siemens Healthineers, Erlangen, Germany), and the slice thickness varied from 3 to 5 mm.

### 2.5. The Generation of the ELVO Reference Standard

Two stroke experts with >10 years of clinical experience generated the ELVO reference standard based on non-contrast CT images, CT angiography, perfusion imaging, and diffusion-weighted MRI. The hemisphere was identified if a patient was diagnosed with a large ELVO. The criteria for ELVO positivity were limited to cases where vascular occlusion from the ICA terminus to the distal M1 segment of the MCA was confirmed on CT angiography, and an acute lesion (infarction) corresponding to the occlusion was identified on perfusion parameter maps such as CBF, CBV, or MTT, or on MR diffusion-weighted images. Core volume criteria or mismatch ratios were not utilized for its classification. If the opinions of the two specialists differed, a consensus was reached through discussion between them.

### 2.6. ELVO Classification Based on Imaging Evaluations

This clinical trial aimed to assess the effectiveness of Heuron ELVO in supporting the clinicians’ identification of ELVO. Therefore, the observer performance method used in previous studies was employed to verify its effectiveness [11,12]. Four readers (one board-certified neurosurgeon, one board-certified emergency medicine specialist, and two neurology residents) performed imaging evaluations. As the principal investigator, an individual senior neurosurgeon gathered the readings of the other investigators and made a final decision based on a majority vote. Such a design was based on the Artificial Intelligence Medical Device Clinical Trial Method Design Guidelines announced by the Korean Ministry of Food and Drug Safety in 2022. The assessment was conducted in two stages. First, an unassisted evaluation was performed by the physicians’ visual grading. After a 2-week washout period, a Heuron ELVO-assisted evaluation was performed by the same investigators using the Heuron ELVO results as a reference, identifying the AI-based presence of ELVO and the involved hemisphere if identified as positive. During the reading, each reader identified the level of suspicion for ELVO using a score of 0–4 (0, no suspicion at all; 1, uncertain but not suspicious; 2, slightly suspicious; 3, suspicious; and 4, highly suspicious). When analyzing the primary endpoint, the 0–1 levels of suspicion were treated as “negative,” and levels “2–4” were treated as positive [13]. The primary investigator made the final decision based on the investigators’ majority-vote level of suspicion. When the results were split into a 2:2 ratio, the primary investigator decided on the final consensus after a personal image review. Both the primary investigator and other readers performed the image reading uniformly. For unassisted reading, they independently assessed non-contrast CT images, and during AI-assisted reading, they referenced AI-provided results while evaluating non-contrast CT scans.

### 2.7. Statistical Analysis

Demographic information such as sex and age was compared between the groups. The chi-square test was used for categorical variables, and an independent *t*-test was used for continuous variables. The statistical analysis of demographics was performed using IBM SPSS Statistics, Version 28 (IBM Corp., Armonk, NY, USA).

The primary endpoint was the sensitivity and specificity of ELVO prediction after Heuron ELVO assistance compared to when only NCCT images were analyzed. Four different analyses were used as secondary endpoints. First, the accuracy of the ELVO classification between the assisted and unassisted analyses was compared. Second, the receiver operating characteristic (ROC) curve and area under the curve (AUROC) values of the classification results were compared between analyses. Third, the differences in sensitivity and specificity between assisted and unassisted analyses for each reader were compared. Finally, the performance of Heuron ELVO in predicting ELVO (reference standard) was validated using sensitivity and specificity analyses.

Significant differences in the indicators between readings analyzed as primary and secondary endpoints were compared using McNemar’s Test (implemented in MedCalc version 20.111, MedCalc Software Ltd., Ostend, Belgium), such as sensitivity, specificity, accuracy, and AUROC. Confidence intervals were derived using the Clopper–Pearson confidence interval method. In general, an AUROC of 0.5 was thought to suggest no discrimination (i.e., the ability to diagnose patients with and without the disease or condition based on the test), 0.7–0.8 was considered acceptable, 0.8–0.9 was considered excellent, and >0.9 was considered outstanding [14]. The interrater reliability of the four readers was analyzed and compared between assisted and unassisted readings using Fleiss’ kappa method [15]. The interpretation of kappa values was as follows: κ < 0 as poor, 0.01–0.20 as slight, 0.21–0.40 as fair, 0.41–0.60 as moderate, 0.61–0.80 as substantial, and 0.81–1.00 as almost perfect agreement [16]. The interrater reliability was analyzed using the Fleiss toolbox (version 2.0.0.0, https://matlab.mathworks.com/open/fileexchange/v1?id=15426 (accessed on 3 August 2023)), which is based on MATLAB (R2023b Update 3, Mathworks, Natick, MA, USA). Please refer to the Supplementary Materials for the formulas of metrics used in the statistical analysis (shown in Table S2).

## 3. Results

### 3.1. Demographics

In the current study, 483 cases were identified through primary screening, and 6 were excluded due to incompatibility with the inclusion/exclusion criteria. Thus, 477 patients were enrolled in this study: 112 patients with ELVO and 365 patients without ELVO. Of the ELVO group, 47.32% (53/112) had large-vessel occlusion in the left hemisphere and 52.68% (59/112) in the right hemisphere. In addition, MCA occlusions accounted for 67.86% of cases (76/112 cases), and intracranial ICA occlusions accounted for 32.14% of cases (36/112 cases). In the non-ELVO group, shown in Table S3., based on the standard disease code (The 8th Korean Standard Classification of Diseases Instruction Manual), 17.2% had cerebral infarction (I63) and cerebrovascular disease (I65, I67), 1.1% had hemorrhagic stroke (I60, I61, I62), 22.2% had a transient ischemic attack (G45.9), 21.6% had vertigo and dizziness (H81.4, R42), 4.4% experienced syncope (R550, R558), and 33.5% had other miscellaneous diagnoses. The mean age was higher in the ELVO group than in the non-ELVO group (68.44 ± 14.31 vs. 58.56 ± 15.62, *p* < 0.01), and the proportion of males was similar in both groups (60.71% vs. 51.51%, *p* = 0.09), while there was a higher rate of comorbid hypertension (66.07% vs. 45.48%, *p* < 0.01) and atrial fibrillation (43.75% vs. 2.74%, *p* < 0.01) in the ELVO group (Table 1).

### 3.2. Primary Endpoint

In the 477 enrolled cases, the sensitivity and specificity were derived from the number of true positives, false negatives, false positives, and true negatives between each evaluation and reference standard. In the unassisted evaluation, the sensitivity and specificity were 75.9% (95% CI: 66.9–83.7%) and 83.0% (95% CI: 78.8–86.7%), with a positive predictive value (PPV) of 57.8% (95% CI: 51.7–63.8%), a negative predictive value (NPV) of 91.8% (95% CI: 89.0–94.0%), and an accuracy of 81.3% (95% CI: 77.6–84.7%). In the Heuron ELVO-assisted evaluation, the sensitivity and specificity were 92.0% (95% CI: 85.3–96.3%) and 92.6% (95% CI: 89.4–95.1%) (Table 2), with a PPV of 79.2% (95% CI: 72.6–84.6%), an NPV of 97.4% (95% CI: 95.3–98.6%), and an accuracy of 92.5% (95% CI: 89.7–94.7%). Both the sensitivity (*p* < 0.01) and specificity (*p* < 0.01) of the assisted evaluation were significantly higher than those of the unassisted evaluation, thereby fulfilling the primary endpoint of the clinical trial.

### 3.3. Secondary Endpoints

**Figure 3:**
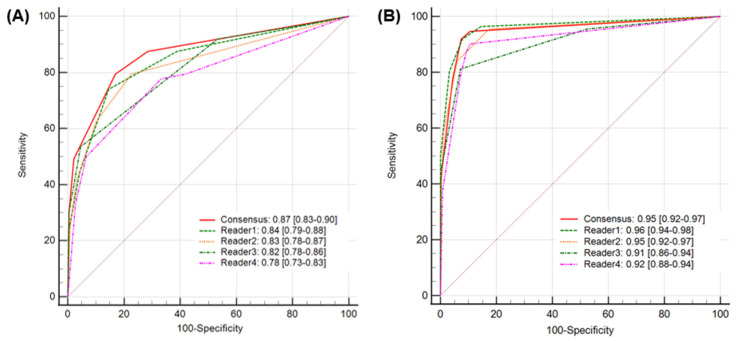
A comparison of AUROCs for AI-assisted analysis (**A**) and unassisted analysis (**B**). The AUROC value of 0.87 (95% CI: 0.83–0.90) for the unassisted evaluation significantly improved to 0.95 (95% CI: 0.92–0.97) for the assisted evaluation. AUROC, area under the receiver operating characteristic curve.

When diagnostic accuracy using Heuron ELVO was evaluated, the calculated accuracy of the unassisted evaluation was 81.3% (95% CI: 77.6–84.7%), while it significantly improved to 92.5% (95% CI: 89.7–94.7%) in assisted evaluations (*p* < 0.01). Regarding the AUROC, a value of 0.87(95% CI: 0.83–0.90) showed excellent discriminative ability in unassisted evaluation, which significantly improved to 0.95 (95% CI: 0.92–0.97), which is considered outstanding in assisted evaluation (*p* = 0.04) (Figure 3). At the individual level, the Heuron ELVO significantly improved the sensitivity and specificity of three out of four readers, respectively (Table 2). The sensitivity and specificity of Heuron ELVO’s standalone performance in predicting ELVO were 88.4% (95% CI: 81.0–93.7) and 91.2% (95% CI: 87.9–93.9), respectively, and showed an AUROC of 0.93, which is classified as outstanding discriminative ability (Table 3) (PPV: 75.6% [95% CI: 68.8–81.3%], NPV: 96.2% [95% CI: 93.9–97.7%], and accuracy: 90.6% [95% CI: 87.6–93.0%]).

### 3.4. Interrater Reliability of Agreement

When the interrater reliability of agreement among readers was analyzed at each stage, the kappa value was 0.27 [95% CI: 0.26–0.28] for the unassisted evaluation, which increased to 0.75 [95% CI: 0.74–0.76] for the Heuron ELVO-assisted evaluation, showing a fair level of interrater agreement in the unassisted stage that improved to a substantial level of agreement in the AI-assisted stage (Table 2).

## 4. Discussion

In the current study, we report on the ability of the Deep Learning-based AI clinical decision support system, Heuron ELVO, to predict ELVO from NCCT images of patients presenting to the emergency room with suspected stroke. While optimized hyperacute stroke imaging protocols facilitate the diagnosis of ELVO, its identification on NCCT images, when possible, is clinically significant, as NCCT is the primary imaging protocol for patients suspected of stroke. This study shows that compared to unassisted evaluation, Heuron ELVO-assisted evaluation improves diagnostic performance in detecting ELVO stroke on NCCT. The Heuron ELVO algorithm resulted in outstanding standalone diagnostic performance. Furthermore, it improved the interrater reliability between readers to a substantial degree.

While some studies have sought to identify ELVO based on NCCT [17,18], the current study is the first clinical trial to show that the NCCT-based identification of ELVO can be improved with AI assistance. Furthermore, with AI assistance, the predictive performance of readers with different backgrounds and expertise can be improved to outstanding levels. The lowest sensitivity and specificity of the assisted evaluation of individual readers were 74% and 91%, respectively, both of which do not differ greatly from previous CT angiography-based AI identifications of large-vessel occlusions [19]. The AUROC of the assisted evaluation was also outstanding, ranging from 0.91 to 0.96 among all four readers in the current trial.

We believe that the AI-assisted identification of ELVO based on NCCT will improve patient outcomes by facilitating interhospital delays and screening for ELVO in patients with minor symptoms in whom angiography was not obtained. In primary hospitals, CTA acquisition may be limited due to concerns about contrast exposure. Furthermore, brain magnetic resonance image-based angiography may be selected as the next imaging modality after NCCT, which can result in significant time losses. AI-supported patient triage based on NCCT images would encourage physicians to transfer potential patients needing reperfusion treatments in a timely manner. Furthermore, there is a growing interest in extending EVT to patients with low neurological severity [20]. Only NCCT would be needed for patients with miscellaneous or minor symptoms, while AI clinical decision support systems may identify ELVO in these patients, specifically those who previously may have been neglected. Overall, the current study does not argue for an EVT patient selection protocol change based on NCCT. Rather, we believe that when CT angiography is not available, AI-supported patient triage based on NCCT images is a practical alternative.

The current study showed a high sensitivity and specificity of 88.39% and 91.23%, respectively, in Heuron ELVO standalone performance when predicting ELVO. Given the difficulty of making direct comparisons between commercially available programs with identical datasets, we reviewed the validation results reported for other products. A previous study reported the diagnostic accuracy of AI-based NCCT ELVO predictions. This study used MethinksLVO, which determines the presence of acute ischemic tissue, chronic lesions, intracranial hemorrhage, and hyperdense clot signs and combines them into a single scalar indicating the presence of LVO. This study reported an AUC of 0.87 for the identification of LVO (with a sensitivity of 83%, a specificity of 71%, a positive predictive value of 79%, and a negative predictive value of 76%) [17]. Another study reported the ability of the RAPID NCCT stroke platform to detect LVO with a sensitivity of 63.5% and specificity of 95.1% [18]. This platform identifies LVO based on HAS and EIC. While the model used in both studies was developed using a similar approach to identify the presence of HAS, EIC, OI, and ELVO, there was a slightly higher predictive performance in the current study. Despite the discrimination of similar biomarkers, the reason for the slightly higher predictive performance in the current study may be the inclusion of eye deviation as a reference. The reported specificity of RAPID NCCT was higher than that observed in our current study. For the validation of RAPID NCCT, the primary causes of false positives in the non-ELVO group were calcified internal carotid arteries or partial volume effects associated with chronic lesions. Heuron ELVO exhibited similar false-positive patterns. However, when human readers interpreted the NCCT images with AI assistance, they could exclude such patterns, suggesting that reader–AI interaction may mitigate some false positives and improve overall specificity in clinical settings. In addition, it may also differ in that the current study only focused on ICA and MCA ELVO (the RAPID NCCT study also limited ELVO patients to anterior circulation). Therefore, errors that could occur during the identification of posterior circulation or anterior cerebral artery ELVO were minimized. Further studies may be needed to determine the contribution of each imaging biomarker element to the overall predictive ability.

Although Heuron ELVO uses a Deep Learning algorithm, it is based on the detection of HAS, EIC, and imaging-based eyeball preponderance. Previously, a visual analysis of the hyperdense artery sign reported a sensitivity of 52% and specificity of 92%. In acute ischemic stroke, HAS indicates a high likelihood of arterial obstruction, but its absence indicates only a 50/50 chance of normal arterial patency [7]. Another study reported a sensitivity and specificity of 67% and 82%, respectively, for detecting ELVO on thin NCCT scans [10]. Radiological eye deviation predicted ELVO with a sensitivity, specificity, and accuracy of 71%, 77.5%, and 84.6%, respectively [8]. Conversely, the predictive value of EIC for ELVO has not been previously evaluated. Theoretically, EIC may be observed in hemispheric or partial ischemic stroke even without ELVO; thus, it cannot be used alone to predict ELVO. In this study, we used EIC along with other variables and believe that it substantially improves the sensitivity of ELVO prediction compared to existing studies using HAS or eye deviations. In this study, we did not identify the specific distributions of EIC that may further point toward ELVO. Further studies are required to address this issue.

## 5. Limitations and Future Work

This clinical trial has some limitations. First, the study population may not represent real-world patients with thrombolysis code activation because this analysis was retrospectively performed based on medical data and imaging protocols. Patients with hemorrhagic stroke were not included in the analysis, and there was a seemingly low number of patients with ischemic stroke in the non-ELVO group. While this is a limitation, it should be noted that a substantial percentage of stroke mimics present to emergency departments [21]. Moreover, as Heuron ELVO is pre-equipped with hemorrhage detection [22], we believe that its diagnostic power results will be comparable in real-world clinical situations. Second, the diagnostic power of clinicians in diagnosing ELVO may have been overestimated because the final adjudication was based on consensus. Nevertheless, we believe that this does not weaken the efficacy of the Heuron ELVO in further assisting clinical decision-making because predictive power was improved during both individual-level evaluation and consensus-based analysis. Bias could be minimized using independent blind assessments or external validation panels. Third, a two-week adjudication interval may not have been sufficient to prevent recall bias, and a longer interval, or analysis by a separate reader, may be ideal in the future. The decision to set the washout period to two weeks in the current study was based on similar, prior research that utilized washout periods from two weeks up to four weeks [23,24]. To further minimize recall bias, we also randomized the order in which the images were presented to the readers, but we could not directly assess the impact of recall bias. Overall, the inherent limitation of the current study lies in its design as a single-center-based retrospective evaluation. Considering the above factors, a prospective multicenter trial evaluating reproducibility across various settings, such as the inclusion of hemorrhagic and posterior circulation stroke, is needed. Furthermore, setting patient classification thresholds for clinical trials should involve a cost–benefit analysis to determine whether reductions in false negatives or false positives should be prioritized in clinical practice, thereby optimizing patient triage. Thess issue will be explored in a prospective study that is being conducted as the next step of this research.

Finally, we acknowledge that a significant portion of the research team is affiliated with Heuron Co. Ltd., which may raise concerns regarding potential conflicts of interest and the risk of bias in study design, data handling, analysis, and interpretation. To mitigate these risks and uphold scientific transparency and objectivity, we implemented the following safeguards. First, although model development was led by Heuron-affiliated researchers, the final validation of the algorithm was conducted using a separate dataset curated and annotated by independent stroke experts unaffiliated with the company. These readers were blinded to both the algorithm output and patient outcomes during annotation. Second, the study followed a predefined analysis plan finalized prior to data collection and analysis. Key endpoints and statistical methods were established as a priori and were not altered post hoc. Third, for both reading stages, all CT image cases were reviewed by board-certified emergency medicine specialists, neurologists, and neurosurgeons who were blinded to the AI results and clinical data during adjudication. Fourth, the data used for model training and testing were locked prior to analysis, and version-controlled audit logs were maintained. Model performance metrics were computed on the locked dataset without retrospective tuning or performance inflation. Fifth, the study protocol was reviewed and approved by an independent IRB, and all research activities adhered to institutional data governance and ethical standards.

In conclusion, Heuron ELVO reliably identified the presence of ELVO on NCCT images, and its assistance significantly improved clinician diagnosis. These findings suggest that Deep Learning algorithm software may assist in shortening the in-hospital process of primary medical centers while facilitating interhospital transfer decisions, resulting in improved treatment outcomes by providing EVT to necessary patients.

## Figures and Tables

**Table 1:** Baseline demographics and comparison of baseline clinical characteristics between the ELVO and control groups.

	ELVO Group (*n* = 112)	Non-ELVO Group (*n* = 365)	*p* Value
Baseline characteristics			
Age, years [Mean ± SD]	68.44 ± 14.31	58.56 ± 15.62	<0.01
Male, *n* (%)	68 (60.71)	188 (51.51)	0.09
Hypertension, *n* (%)	74 (66.07)	166 (45.48)	<0.01
Diabetes mellitus, *n* (%)	25 (22.32)	92 (25.21)	0.53
Cardiac disease, *n* (%)	6 (5.36)	7 (1.92)	0.05
Dyslipidemia, *n* (%)	23 (20.54)	81 (22.19)	0.71
Atrial fibrillation, *n* (%)	49 (43.75)	10 (2.74)	<0.01
Smoking, *n* (%)	35 (31.25)	81 (22.19)	0.05
IV thrombolysis, *n* (%)	71 (63.39)	-	
IA thrombectomy, *n* (%)	69 (61.61)	-	
**ELVO hemisphere information according to the reference standard**			
	Left hemisphere, *n* (%)	Right hemisphere, *n* (%)	
ELVO-positive (*n* = 112)	53 (47.32)	59 (52.68)	

ELVO, emergent large vessel occlusion; IV, intravenous; IA, intra-arterial; SD, standard deviation.

**Table 2:** A comparison of ELVO classification performance considering sensitivity, specificity, and area under the ROC curve (AUROC) of unassisted and AI-assisted analysis for the main adjudication and individual-investigator-level analysis.

	Sensitivity			Specificity			AUROC		
Consensus-level analysis									
	Unassisted stage	AI-assisted stage	*p* value	Unassisted stage	AI-assisted stage	*p* value	Unassisted stage	AI-assisted stage	*p* value
%, [95% CI]	75.9 (85/112), [66.9–83.7]	92.0 (103/112), [85.3–96.3]	<0.01	83.0 (303/365), [78.8–86.7]	92.6 (338/365), [89.4–95.1]	<0.01	0.87, [0.83–0.90]	0.95, [0.92–0.97]	0.04
**Individual-level analysis %, [95% CI]**									
Reader 1	68.8 (77/112), [59.3–77.2]	90.2 (101/112), [83.1–95.0]	<0.01	85.5 (312/365), [81.4–88.9]	92.1 (336/365), [88.8–94.6]	<0.01	0.84, [0.79–0.88]	0.96, [0.94–0.98]	0.02
Reader 2	58.0 (65/112), [48.3–67.3]	81.3 (91/112), [72.8–88.0]	<0.01	89.86 (328/365), [86.3–92.8]	93.97 (343/365), [91.0–96.2]	0.04	0.83, [0.78–0.87]	0.95, [0.92–0.97]	0.02
Reader 3	85.7 (96/112), [77.8–91.6]	79.5 (89/112), [70.8–86.5]	0.26	46.6 (170/365), [41.4–51.8]	92.9 (339/365), [89.7–95.3]	<0.01	0.82, [0.78–0.86]	0.91, [0.86–0.94]	0.11
Reader 4	57.1 (64/112), [47.5–66.5]	74.1 (83/112), [65.0–81.9]	<0.01	66.9 (244/365), [61.8–71.7]	90.7 (331/365), [87.2–93.5]	< 0.01	0.78, [0.73–0.83]	0.92, [0.88–0.94]	0.02
**Interrater reliability of agreement**									
**Fleiss’ kappa**				**Unassisted stage**			**AI-assisted stage**		
**κ [95% CI]**				**0.27 [0.26–0.28]**			**0.75 [0.74–0.76]**		

ELVO, emergent large vessel occlusion; AUROC, area under the receiver operative characteristics; CI, confidence interval; AI, artificial intelligence.

**Table 3:** The standalone performance of Heuron ELVO in the prediction of anterior circulation ELVO.

ELVO Classification	Sensitivity % [95% CI]	Specificity % [95% CI]	AUROC [95% CI]
Heuron ELVO	88.4 (99/112) [81.0–93.7]	91.2 (333/365) [87.9–93.9]	0.93 [0.90–0.96]
RAPID-NCCT	63.5 (73/115) [54.4–71.7]	95.1 (98/103) [89.1–97.9]	NA
MethinkLVO	83.2 (685/823) [80.5–85.7]	71.3 (449/630) [67.6–74.8]	0.87

ELVO, emergent large vessel occlusion; CI, confidence interval; AUROC, area under the receiver operative characteristics.

## Data Availability

The data supporting the findings of this study are available from the corresponding authors upon request.

## Supplementary Materials

The following supporting information can be downloaded at https://www.mdpi.com/article/10.3390/jcm14134635/s1. Figure S1. ROC curve and bar graph of model validation (5-fold cross validation, Mean ensemble, Threshold = 0.5). Table S1. Confusion metrics, and performance of model validation (5-fold cross validation, Mean ensemble, Threshold = 0.5). Table S2. Formulas of statistical metrics. Table S3. Confirmed Diagnosis in Non-ELVO Group with readers’ consensus according to unassisted and AI-assisted reading.
