# Supplementary material for: Using a Deep Learning-Based Decision Support System to Predict Emergent Large Vessel Occlusion Using Non-Contrast Computed Tomography
Source: J Clin Med. 2025 Jun 30;14(13):4635. doi: 10.3390/jcm14134635 (PMC12250463; doi:10.3390/jcm14134635)
Supplement: Supplementary file 1 [file jcm-14-04635-s001.zip › jcm-3681787-supplementary.pdf]

Using a Deep Learning-Based Decision Support System to Predict Emergent Large Vessel Occlusion Using Non-Contrast Computed Tomography

Seong-Joon Lee,[1]* Dohyun Kim,[2]* Dae Han Choi,[3] Yong Su Lim,[4] Gyuha Park,[2] Sumin Jung,[2] Soohwa Song,[2] Ji Man Hong,[1] Dong Hoon Shin,[2,5] Myeong Jin Kim,[3]† Jin Soo Lee[1]†

[1]Department of Neurology, Ajou University School of Medicine, 164, World Cup-ro, Yeongtong-gu, Suwon-si, Gyeonggi-do 16499, South Korea

[2]Research Division, Heuron Co., Ltd., 10F, C, 150 Yeongdeungpo-ro, Yeongdeungpo-gu, Seoul 07282, South Korea

[3]Department of Neurosurgery, Gachon University College of Medicine, 38-13, Dokjeom-ro 3beon-gil, Namdong-gu, Incheon 21565, South Korea.

[4]Department of Emergency Medicine, Gachon University College of Medicine, 38-13, Dokjeom-ro 3beon-gil, Namdong-gu, Incheon 21565, South Korea.

[5]Department of Neurology, Gachon University College of Medicine, 38-13, Dokjeom-ro 3beon-gil, Namdong-gu, Incheon 21565, South Korea.

*These authors contributed equally as the first authors of the current paper

† Correspondence:

Myeong Jin Kim. MD, PhD

Department of Neurosurgery, Gachon University College of Medicine; 38-13, Dokjeom-ro 3beon-gil, Namdong-gu, Incheon 21565, South Korea.

Tel: +82-32-460-3304

Fax: +83-32-460-3899

E-mail: skymedi@gachon.ac.kr.

Jin Soo Lee, MD, PhD

Department of Neurology, Ajou University School of Medicine; 164, World Cup-ro, Yeongtong-gu, Suwon-si, Gyeonggi-do 16499, South Korea

Tel: +82-31-219-5175

Fax: +82-31-219-5178

E-mail: jinsoo22@gmail.com

Supplementary Figure S1. ROC curve and bar graph of model validation (5-fold cross validation, Mean ensemble, Threshold = 0.5)

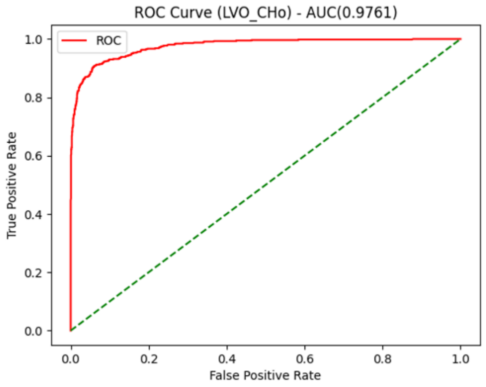

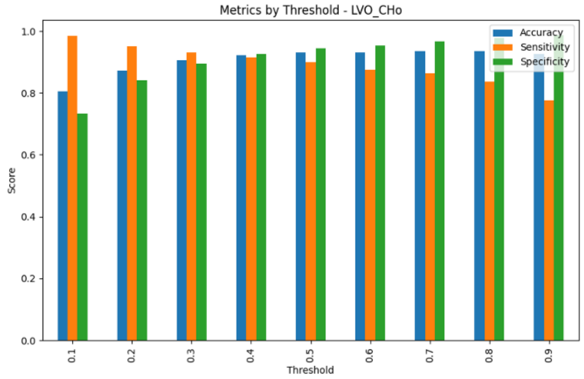

Supplementary Table S1. Confusion metrics, and performance of model validation (5-fold cross validation, Mean ensemble, Threshold = 0.5)

5-fold CV total count	GT-Positive	GT-Negative
Prediction-Positive	241	43
Prediction-Negative	21	609

- Sensitivity = 0.9198
- Specificity = 0.9340
- Accuracy = 0.9300
- AUROC = 0.9761

Supplementary Table S2. Formulas of statistical metrics

Metrics	Formula
Sensitivity	$\frac{True\ Positive\#}{True\ Positive\# + False\ Negative\#}$
Specificit y	$\frac{True\ Negative\#}{True\ Negative\# + False\ Positive\#}$
PPV*	$\frac{True\ Positive\#}{True\ Positive\# + False\ Positive\#}$
NPV**	$\frac{True\ Negative\#}{True\ Negative\# + False\ Negative\#}$
Accuracy	$\frac{True\ Positive\# + True\ Negative\#}{True\ Positive\# + False\ Negative\# + True\ Negative\# + False\ Positive\#}$

** PPV: Positive Prediction Value, **NPV: Negative Prediction Value*

Supplementary Table S3. Confirmed Diagnosis in Non-ELVO Group with readers' consensus according to unassisted and AI-assisted reading

Screening No.	Disease Code (Disease Name) in Non-ELVO Group	Consensus Results of Unassisted Reading (P/N)	Consensus Results of AI-assisted Reading (P/N)
S114	I602 (Diseases of the circulatory system - Subarachnoid haemorrhage from anterior communicating artery)	P	N
S115	T601 (Injury, poisoning, and other specific consequences of exogenous factors - Toxic effects of halogenated insecticides)	N	N
S116	G450 (Diseases of the nervous system - Vertebro-basilar artery syndrome)	N	N
S117	I6330 (Diseases of the circulatory system - Cerebral infarction due to thrombosis in middle cerebral artery)	P	N
S118	G459 (Diseases of the nervous system - Transient cerebral ischemic attacks and related syndromes)	N	N
S119	G459 (Diseases of the nervous system - Transient cerebral ischemic attacks and related syndromes)	N	N
S120	G459 (Diseases of the nervous system - Transient cerebral ischemic attacks and related syndromes)	N	P
S121	G459 (Diseases of the nervous system - Transient cerebral ischemic attacks and related syndromes)	N	N
S122	G459 (Diseases of the nervous system - Transient cerebral ischemic attacks and related syndromes)	N	N
S123	B004 (Certain infectious and parasitic diseases - Herpesviral encephalitis)	N	P
S124	H814 (Diseases of the ear and mastoid - Vertigo of central origin)	N	N
S125	H814 (Diseases of the ear and mastoid - Vertigo of central origin)	N	N
S126	I480 (Diseases of the circulatory system - Paroxysmal atrial fibrillation)	N	N
S127	H341 (Diseases of the eye and eye appendages - Central retinal artery occlusion)	N	P
S128	I6330 (Diseases of the circulatory system - Cerebral infarction due to thrombosis in middle cerebral artery)	P	N
S129	I638 (Diseases of the circulatory system - Other cerebral infarction)	P	N
S130	G20 (Diseases of the nervous system - Parkinson's disease)	N	N
S131	G20 (Diseases of the nervous system - Parkinson's disease)	N	N
S132	H814 (Diseases of the ear and mastoid - Vertigo of central origin)	N	P
S133	G454 (Diseases of the nervous system - Transient global amnesia)	N	P
S134	R42 (Symptoms, signs, and clinical and laboratory abnormalities not otherwise specified - Dizziness)	N	N
S135	G934 (Diseases of the nervous system - Encephalopathy, unspecified)	N	N
S136	E1168 (Endocrine, nutritional and metabolic diseases - Type 2 diabetes mellitus, with multiple complications)	N	P
S137	G510 (Diseases of the nervous system - Bell's palsy)	N	N

S138	G431 (Diseases of the nervous system - Migraine with aura)	N	N
S139	I6339 (Diseases of the circulatory system - Cerebral infarction due to thrombosis of unspecified cerebral arteries)	N	N
S140	G459 (Diseases of the nervous system - Transient cerebral ischemic attacks and related syndromes)	P	P
S141	I209 (Diseases of the circulatory system - Angina pectoris, unspecified)	N	N
S142	G459 (Diseases of the nervous system - Transient cerebral ischemic attacks and related syndromes)	N	N
S143	I679 (Diseases of the circulatory system - Cerebrovascular disease, unspecified)	N	N
S144	R51 (Symptoms, signs, and clinical and laboratory abnormalities not otherwise specified - Headache)	N	N
S145	H814 (Diseases of the ear and mastoid - Vertigo of central origin)	N	N
S146	A170 (Certain infectious and parasitic diseases - Tuberculous meningitis)	N	N
S147	H812 (Diseases of the ear and mastoid - Vestibular neuronitis)	N	P
S148	F103 (Mental and behavioral disorders - Withdrawal state of alcohol)	N	N
S149	I639 (Diseases of the circulatory system - Cerebral infarction, unspecified)	P	N
S150	M5422 (Musculoskeletal system and connective tissue diseases - Cervicalgia, cervical region)	N	N
S151	H814 (Diseases of the ear and mastoid - Vertigo of central origin)	N	N
S152	N185 (Diseases of the urogenital system - Chronic kidney disease, stage 5)	P	N
S153	I639 (Diseases of the circulatory system - Cerebral infarction, unspecified)	N	N
S154	R51 (Symptoms, signs, and clinical and laboratory abnormalities not otherwise specified - Headache)	N	N
S155	M512 (Musculoskeletal system and connective tissue diseases - Other specified intervertebral disc displacement)	N	N
S156	I6339 (Diseases of the circulatory system - Cerebral infarction due to thrombosis of unspecified cerebral arteries)	P	N
S157	M501 (Musculoskeletal system and connective tissue diseases - Cervical disc disorder with radiculopathy)	N	N
S158	G454 (Diseases of the nervous system - Transient global amnesia)	N	P
S159	I652 (Diseases of the circulatory system - Occlusion and stenosis of carotid artery)	N	N
S160	I611 (Diseases of the circulatory system - Intracerebral haemorrhage in hemisphere, cortical)	P	P
S161	R42 (Symptoms, signs, and clinical and laboratory abnormalities not otherwise specified - Dizziness)	N	N
S162	H532 (Diseases of the eye and eye appendages - Double vision)	N	P
S163	I6332 (Diseases of the circulatory system - Cerebral infarction due to thrombosis of anterior cerebral artery)	P	P
S164	I6300 (Diseases of the circulatory system - Cerebral infarction due to thrombosis of vertebral artery)	N	P

S165	I639 (Diseases of the circulatory system - Cerebral infarction, unspecified)	P	N
S166	R073 (Symptoms, signs, and clinical and laboratory abnormalities not otherwise specified - Other chest pain)	N	N
S167	G510 (Diseases of the nervous system - Bell's palsy)	N	P
S168	I639 (Diseases of the circulatory system - Cerebral infarction, unspecified)	P	P
S169	G459 (Diseases of the nervous system - Transient cerebral ischemic attacks and related syndromes)	N	N
S170	I639 (Diseases of the circulatory system - Cerebral infarction, unspecified)	N	N
S171	G430 (Diseases of the nervous system - Migraine without aura)	N	N
S172	C20 (Neoplasm - Malignant neoplasm of the rectum)	N	N
S173	I200 (Diseases of the circulatory system - Unstable angina)	P	N
S174	S065 (Injury, poisoning, and other specific consequences of exogenous factors - Traumatic subdural haemorrhage)	N	N
S175	I639 (Diseases of the circulatory system - Cerebral infarction, unspecified)	N	N
S176	H814 (Diseases of the ear and mastoid - Vertigo of central origin)	N	N
S177	R558 (Symptoms, signs, and clinical and laboratory abnormalities not otherwise specified - Other syncope and collapse)	P	N
S178	G459 (Diseases of the nervous system - Transient cerebral ischemic attacks and related syndromes)	N	N
S179	G4090 (Diseases of the nervous system - Epilepsy, unspecified without intractable epilepsy)	N	N
S180	R471 (Symptoms, signs, and clinical and laboratory abnormalities not otherwise specified - Dysarthria and anarthria)	N	N
S181	H814 (Diseases of the ear and mastoid - Vertigo of central origin)	N	N
S182	I639 (Diseases of the circulatory system - Cerebral infarction, unspecified)	N	P
S183	I6338 (Diseases of the circulatory system - Cerebral infarction due to thrombosis of other cerebral artery)	N	N
S184	F103 (Mental and behavioral disorders - Withdrawal state of alcohol)	N	P
S185	R558 (Symptoms, signs, and clinical and laboratory abnormalities not otherwise specified - Other syncope and collapse)	N	N
S186	G459 (Diseases of the nervous system - Transient cerebral ischemic attacks and related syndromes)	N	N
S187	G20 (Diseases of the nervous system - Parkinson's disease)	N	N
S188	I639 (Diseases of the circulatory system - Cerebral infarction, unspecified)	N	N
S189	G442 (Diseases of the nervous system - Tension-type headache)	N	N
S190	I613 (Diseases of the circulatory system - Intracerebral haemorrhage in brain stem)	P	N
S191	I639 (Diseases of the circulatory system - Cerebral infarction, unspecified)	P	N

S192	G513 (Diseases of the nervous system - Clonic hemifacial spasm)	N	P
S193	I639 (Diseases of the circulatory system - Cerebral infarction, unspecified)	P	P
S194	E162 (Endocrine, nutritional and metabolic diseases - Hypoglycemia, unspecified)	N	N
S195	H814 (Diseases of the ear and mastoid - Vertigo of central origin)	N	P
S196	I639 (Diseases of the circulatory system - Cerebral infarction, unspecified)	P	N
S197	I6301 (Diseases of the circulatory system - Cerebral infarction due to thrombosis of basilar artery)	P	N
S198	G459 (Diseases of the nervous system - Transient cerebral ischemic attacks and related syndromes)	N	N
S199	G459 (Diseases of the nervous system - Transient cerebral ischemic attacks and related syndromes)	N	N
S200	I6338 (Diseases of the circulatory system - Cerebral infarction due to thrombosis of other cerebral artery)	P	P
S201	H814 (Diseases of the ear and mastoid - Vertigo of central origin)	N	N
S202	G20 (Diseases of the nervous system - Parkinson's disease)	N	N
S203	G454 (Diseases of the nervous system - Transient global amnesia)	P	N
S204	R558 (Symptoms, signs, and clinical and laboratory abnormalities not otherwise specified - Other syncope and collapse)	N	N
S205	H814 (Diseases of the ear and mastoid - Vertigo of central origin)	N	P
S206	G4090 (Diseases of the nervous system - Epilepsy, unspecified without intractable epilepsy)	N	N
S207	R42 (Symptoms, signs, and clinical and laboratory abnormalities not otherwise specified - Dizziness)	N	N
S208	I638 (Diseases of the circulatory system - Other cerebral infarction)	N	N
S209	G4090 (Diseases of the nervous system - Epilepsy, unspecified without intractable epilepsy)	N	N
S210	H814 (Diseases of the ear and mastoid - Vertigo of central origin)	N	N
S211	I639 (Diseases of the circulatory system - Cerebral infarction, unspecified)	P	N
S212	H812 (Diseases of the ear and mastoid - Vestibular neuronitis)	N	N
S213	I671 (Diseases of the circulatory system - Cerebral aneurysm, nonruptured)	N	P
S214	R558 (Symptoms, signs, and clinical and laboratory abnormalities not otherwise specified - Other syncope and collapse)	N	N
S215	R42 (Symptoms, signs, and clinical and laboratory abnormalities not otherwise specified - Dizziness)	N	N
S216	I620 (Diseases of the circulatory system - Nontraumatic extradural haemorrhage)	N	N
S217	I639 (Diseases of the circulatory system - Cerebral infarction, unspecified)	N	N
S218	S0640 (Injury, poisoning, and other specific consequences of exogenous factors - Extradural haemorrhage, without open intracranial wound)	P	N

S219	G459 (Diseases of the nervous system - Transient cerebral ischemic attacks and related syndromes)	N	N
S220	G454 (Diseases of the nervous system - Transient global amnesia)	N	N
S221	I650 (Diseases of the circulatory system - Occlusion and stenosis of vertebral artery)	N	N
S223	F449 (Mental and behavioral disorders - Dissociative disorder, unspecified)	N	N
S224	H814 (Diseases of the ear and mastoid - Vertigo of central origin)	N	N
S225	I639 (Diseases of the circulatory system - Cerebral infarction, unspecified)	N	N
S226	I653 (Diseases of the circulatory system - Occlusion and stenosis of multiple and bilateral precerbral arteries)	N	P
S227	I638 (Diseases of the circulatory system - Other cerebral infarction)	P	N
S228	H814 (Diseases of the ear and mastoid - Vertigo of central origin)	N	N
S229	I639 (Diseases of the circulatory system - Cerebral infarction, unspecified)	N	N
S230	G039 (Diseases of the nervous system - Meningitis, unspecified)	N	N
S231	I639 (Diseases of the circulatory system - Cerebral infarction, unspecified)	N	N
S232	I639 (Diseases of the circulatory system - Cerebral infarction, unspecified)	N	P
S233	H814 (Diseases of the ear and mastoid - Vertigo of central origin)	P	N
S234	H814 (Diseases of the ear and mastoid - Vertigo of central origin)	N	N
S235	G009 (Diseases of the nervous system - Bacterial meningitis, unspecified)	N	N
S237	I6301 (Diseases of the circulatory system - Cerebral infarction due to thrombosis of basilar artery)	P	N
S238	I639 (Diseases of the circulatory system - Cerebral infarction, unspecified)	N	P
S239	I639 (Diseases of the circulatory system - Cerebral infarction, unspecified)	P	N
S240	I639 (Diseases of the circulatory system - Cerebral infarction, unspecified)	P	N
S241	H814 (Diseases of the ear and mastoid - Vertigo of central origin)	N	N
S242	I639 (Diseases of the circulatory system - Cerebral infarction, unspecified)	N	N
S243	I639 (Diseases of the circulatory system - Cerebral infarction, unspecified)	N	N
S244	I639 (Diseases of the circulatory system - Cerebral infarction, unspecified)	N	P
S246	G459 (Diseases of the nervous system - Transient cerebral ischemic attacks and related syndromes)	N	N
S247	I639 (Diseases of the circulatory system - Cerebral infarction, unspecified)	N	N
S248	H812 (Diseases of the ear and mastoid - Vestibular neuronitis)	N	P
S249	G459 (Diseases of the nervous system - Transient cerebral ischemic attacks and related syndromes)	P	P

S250	H814 (Diseases of the ear and mastoid - Vertigo of central origin)	P	N
S251	R448 (Symptoms, signs, and clinical and laboratory abnormalities not otherwise specified - Other and unspecified symptoms and signs involving general sensations and perceptions)	N	P
S252	I638 (Diseases of the circulatory system - Other cerebral infarction)	P	N
S253	R42 (Symptoms, signs, and clinical and laboratory abnormalities not otherwise specified - Dizziness)	N	N
S254	H814 (Diseases of the ear and mastoid - Vertigo of central origin)	N	N
S255	I639 (Diseases of the circulatory system - Cerebral infarction, unspecified)	N	N
S256	R42 (Symptoms, signs, and clinical and laboratory abnormalities not otherwise specified - Dizziness)	N	N
S257	I652 (Diseases of the circulatory system - Occlusion and stenosis of carotid artery)	N	N
S258	G4090 (Diseases of the nervous system - Epilepsy, unspecified without intractable epilepsy)	N	N
S259	G459 (Diseases of the nervous system - Transient cerebral ischemic attacks and related syndromes)	N	P
S260	I6300 (Diseases of the circulatory system - Cerebral infarction due to thrombosis of vertebral artery)	N	N
S261	R42 (Symptoms, signs, and clinical and laboratory abnormalities not otherwise specified - Dizziness)	N	N
S262	I6338 (Diseases of the circulatory system - Cerebral infarction due to thrombosis of other cerebral artery)	N	N
S263	H814 (Diseases of the ear and mastoid - Vertigo of central origin)	N	P
S264	I638 (Diseases of the circulatory system - Other cerebral infarction)	P	P
S265	G442 (Diseases of the nervous system - Tension-type headache)	N	N
S266	R42 (Symptoms, signs, and clinical and laboratory abnormalities not otherwise specified - Dizziness)	N	N
S267	H814 (Diseases of the ear and mastoid - Vertigo of central origin)	N	N
S268	H814 (Diseases of the ear and mastoid - Vertigo of central origin)	N	P
S269	I639 (Diseases of the circulatory system - Cerebral infarction, unspecified)	N	N
S270	I639 (Diseases of the circulatory system - Cerebral infarction, unspecified)	N	N
S271	R72 (Symptoms, signs, and clinical and laboratory abnormalities not otherwise specified - Abnormality of white blood cells, NEC)	N	P
S272	I639 (Diseases of the circulatory system - Cerebral infarction, unspecified)	N	N
S273	H814 (Diseases of the ear and mastoid - Vertigo of central origin)	N	N
S274	G9388 (Diseases of the nervous system - Other specified disorders of brain)	N	P
S275	H814 (Diseases of the ear and mastoid - Vertigo of central origin)	N	N
S276	I639 (Diseases of the circulatory system - Cerebral infarction, unspecified)	N	P

S277	G459 (Diseases of the nervous system - Transient cerebral ischemic attacks and related syndromes)	N	N
S278	I639 (Diseases of the circulatory system - Cerebral infarction, unspecified)	P	N
S279	I639 (Diseases of the circulatory system - Cerebral infarction, unspecified)	P	N
S280	R558 (Symptoms, signs, and clinical and laboratory abnormalities not otherwise specified - Other syncope and collapse)	N	N
S281	R568 (Symptoms, signs, and clinical and laboratory abnormalities not otherwise specified - Other and unspecified convulsions)	N	N
S282	H814 (Diseases of the ear and mastoid - Vertigo of central origin)	N	P
S283	G459 (Diseases of the nervous system - Transient cerebral ischemic attacks and related syndromes)	N	N
S284	I639 (Diseases of the circulatory system - Cerebral infarction, unspecified)	N	P
S285	H814 (Diseases of the ear and mastoid - Vertigo of central origin)	N	N
S286	I638 (Diseases of the circulatory system - Other cerebral infarction)	N	N
S287	I639 (Diseases of the circulatory system - Cerebral infarction, unspecified)	N	N
S288	R42 (Symptoms, signs, and clinical and laboratory abnormalities not otherwise specified - Dizziness)	N	N
S289	H814 (Diseases of the ear and mastoid - Vertigo of central origin)	N	N
S290	G510 (Diseases of the nervous system - Bell’s palsy)	N	P
S291	I639 (Diseases of the circulatory system - Cerebral infarction, unspecified)	N	N
S292	I639 (Diseases of the circulatory system - Cerebral infarction, unspecified)	N	N
S293	H814 (Diseases of the ear and mastoid - Vertigo of central origin)	N	N
S294	I638 (Diseases of the circulatory system - Other cerebral infarction)	N	N
S295	R471 (Symptoms, signs, and clinical and laboratory abnormalities not otherwise specified - Dysarthria and anarthria)	N	P
S296	I639 (Diseases of the circulatory system - Cerebral infarction, unspecified)	N	N
S298	G441 (Diseases of the nervous system - Vascular headache, NEC)	N	N
S299	G431 (Diseases of the nervous system - Migraine with aura)	N	N
S300	H812 (Diseases of the ear and mastoid - Vestibular neuronitis)	N	N
S301	R51 (Symptoms, signs, and clinical and laboratory abnormalities not otherwise specified - Headache)	N	N
S302	G459 (Diseases of the nervous system - Transient cerebral ischemic attacks and related syndromes)	N	N
S303	G459 (Diseases of the nervous system - Transient cerebral ischemic attacks and related syndromes)	N	N
S304	F100 (Mental and behavioral disorders - Acute intoxication due to use of alcohol)	P	P
S305	I639 (Diseases of the circulatory system - Cerebral infarction, unspecified)	P	P

S306	I6330 (Diseases of the circulatory system - Cerebral infarction due to thrombosis in middle cerebral artery)	N	N
S307	U129 (Special purpose code - COVID-19 vaccines causing adverse effects in therapeutic use, unspecified)	N	P
S308	H814 (Diseases of the ear and mastoid - Vertigo of central origin)	N	P
S309	H814 (Diseases of the ear and mastoid - Vertigo of central origin)	N	N
S310	G459 (Diseases of the nervous system - Transient cerebral ischemic attacks and related syndromes)	N	N
S311	R471 (Symptoms, signs, and clinical and laboratory abnormalities not otherwise specified - Dysarthria and anarthria)	N	P
S312	G454 (Diseases of the nervous system - Transient global amnesia)	N	N
S313	G459 (Diseases of the nervous system - Transient cerebral ischemic attacks and related syndromes)	N	N
S314	G459 (Diseases of the nervous system - Transient cerebral ischemic attacks and related syndromes)	P	N
S315	G459 (Diseases of the nervous system - Transient cerebral ischemic attacks and related syndromes)	N	N
S316	Z721 (Factors affecting health status and access to health services - Alcohol use)	N	P
S317	R558 (Symptoms, signs, and clinical and laboratory abnormalities not otherwise specified - Other syncope and collapse)	N	P
S318	G459 (Diseases of the nervous system - Transient cerebral ischemic attacks and related syndromes)	N	N
S319	G459 (Diseases of the nervous system - Transient cerebral ischemic attacks and related syndromes)	N	P
S320	H814 (Diseases of the ear and mastoid - Vertigo of central origin)	N	N
S321	R42 (Symptoms, signs, and clinical and laboratory abnormalities not otherwise specified - Dizziness)	N	P
S322	G459 (Diseases of the nervous system - Transient cerebral ischemic attacks and related syndromes)	P	N
S323	H8138 (Diseases of the ear and mastoid - Other peripheral vertigo)	N	P
S324	R558 (Symptoms, signs, and clinical and laboratory abnormalities not otherwise specified - Other syncope and collapse)	N	N
S325	G459 (Diseases of the nervous system - Transient cerebral ischemic attacks and related syndromes)	N	P
S326	H814 (Diseases of the ear and mastoid - Vertigo of central origin)	N	N
S327	R42 (Symptoms, signs, and clinical and laboratory abnormalities not otherwise specified - Dizziness)	P	N
S328	H814 (Diseases of the ear and mastoid - Vertigo of central origin)	N	N
S329	H819 (Diseases of the ear and mastoid - Disorder of vestibular function, unspecified)	N	N
S330	G459 (Diseases of the nervous system - Transient cerebral ischemic attacks and related syndromes)	N	N
S331	G459 (Diseases of the nervous system - Transient cerebral ischemic attacks and related syndromes)	N	N
S332	R410 (Symptoms, signs, and clinical and laboratory abnormalities not otherwise specified - Disorientation, unspecified)	P	N

S333	F410 (Mental and behavioral disorders - Panic disorder)	N	N
S334	H814 (Diseases of the ear and mastoid - Vertigo of central origin)	N	N
S335	G459 (Diseases of the nervous system - Transient cerebral ischemic attacks and related syndromes)	N	P
S336	R208 (Symptoms, signs, and clinical and laboratory abnormalities not otherwise specified - Other and unspecified disturbances of skin sensation)	N	N
S337	R42 (Symptoms, signs, and clinical and laboratory abnormalities not otherwise specified - Dizziness)	N	N
S338	R42 (Symptoms, signs, and clinical and laboratory abnormalities not otherwise specified - Dizziness)	P	N
S339	G629 (Diseases of the nervous system - Polyneuropathy, unspecified)	P	P
S340	G459 (Diseases of the nervous system - Transient cerebral ischemic attacks and related syndromes)	N	P
S341	G454 (Diseases of the nervous system - Transient global amnesia)	N	N
S342	H814 (Diseases of the ear and mastoid - Vertigo of central origin)	N	N
S343	R51 (Symptoms, signs, and clinical and laboratory abnormalities not otherwise specified - Headache)	N	N
S344	G459 (Diseases of the nervous system - Transient cerebral ischemic attacks and related syndromes)	N	N
S345	G4090 (Diseases of the nervous system - Epilepsy, unspecified without intractable epilepsy)	P	P
S346	G454 (Diseases of the nervous system - Transient global amnesia)	N	N
S347	H8138 (Diseases of the ear and mastoid - Other peripheral vertigo)	N	P
S348	G459 (Diseases of the nervous system - Transient cerebral ischemic attacks and related syndromes)	N	P
S349	H814 (Diseases of the ear and mastoid - Vertigo of central origin)	N	N
S350	G459 (Diseases of the nervous system - Transient cerebral ischemic attacks and related syndromes)	N	P
S351	R550 (Symptoms, signs, and clinical and laboratory abnormalities not otherwise specified - Vasovagal syncope)	N	P
S352	G459 (Diseases of the nervous system - Transient cerebral ischemic attacks and related syndromes)	N	P
S353	G459 (Diseases of the nervous system - Transient cerebral ischemic attacks and related syndromes)	P	N
S354	S141 (Injury, poisoning, and other specific consequences of exogenous factors - Other and unspecified injuries of cervical spinal cord)	N	P
S355	G459 (Diseases of the nervous system - Transient cerebral ischemic attacks and related syndromes)	N	N
S356	F430 (Mental and behavioral disorders - Acute stress reaction)	N	N
S357	H814 (Diseases of the ear and mastoid - Vertigo of central origin)	N	N
S358	H814 (Diseases of the ear and mastoid - Vertigo of central origin)	N	N
S359	G439 (Diseases of the nervous system - Migraine, unspecified)	N	N

S360	G20 (Diseases of the nervous system - Parkinson's disease)	N	P
S361	R471 (Symptoms, signs, and clinical and laboratory abnormalities not otherwise specified - Dysarthria and anarthria)	N	N
S362	G454 (Diseases of the nervous system - Transient global amnesia)	N	N
S363	R558 (Symptoms, signs, and clinical and laboratory abnormalities not otherwise specified - Other syncope and collapse)	N	N
S364	H814 (Diseases of the ear and mastoid - Vertigo of central origin)	N	N
S365	G459 (Diseases of the nervous system - Transient cerebral ischemic attacks and related syndromes)	N	N
S366	G459 (Diseases of the nervous system - Transient cerebral ischemic attacks and related syndromes)	N	N
S367	R413 (Symptoms, signs, and clinical and laboratory abnormalities not otherwise specified - Other amnesia)	N	N
S368	G528 (Diseases of the nervous system - Disorders of other specified cranial nerves)	N	N
S369	H8138 (Diseases of the ear and mastoid - Other peripheral vertigo)	N	N
S370	R558 (Symptoms, signs, and clinical and laboratory abnormalities not otherwise specified - Other syncope and collapse)	N	P
S371	G459 (Diseases of the nervous system - Transient cerebral ischemic attacks and related syndromes)	N	P
S372	G629 (Diseases of the nervous system - Polyneuropathy, unspecified)	N	P
S373	G459 (Diseases of the nervous system - Transient cerebral ischemic attacks and related syndromes)	N	N
S374	G231 (Diseases of the nervous system - Progressive supranuclear ophthalmoplegia)	N	N
S375	F067 (Mental and behavioral disorders - Mild cognitive disorder)	N	N
S376	G459 (Diseases of the nervous system - Transient cerebral ischemic attacks and related syndromes)	N	N
S377	G459 (Diseases of the nervous system - Transient cerebral ischemic attacks and related syndromes)	N	P
S378	H814 (Diseases of the ear and mastoid - Vertigo of central origin)	N	P
S379	F101 (Mental and behavioral disorders - Harmful use of alcohol)	N	N
S380	H814 (Diseases of the ear and mastoid - Vertigo of central origin)	N	N
S381	H814 (Diseases of the ear and mastoid - Vertigo of central origin)	P	
S382	G459 (Diseases of the nervous system - Transient cerebral ischemic attacks and related syndromes)	P	N
S383	Y471 (External causes of morbidity and death - Benzodiazepine)	P	N
S384	G459 (Diseases of the nervous system - Transient cerebral ischemic attacks and related syndromes)	N	N
S385	R42 (Symptoms, signs, and clinical and laboratory abnormalities not otherwise specified - Dizziness)	N	N
S386	F101 (Mental and behavioral disorders - Harmful use of alcohol)	N	N

S387	H814 (Diseases of the ear and mastoid - Vertigo of central origin)	N	N
S388	H814 (Diseases of the ear and mastoid - Vertigo of central origin)	N	N
S389	H814 (Diseases of the ear and mastoid - Vertigo of central origin)	N	N
S390	G459 (Diseases of the nervous system - Transient cerebral ischemic attacks and related syndromes)	P	P
S391	G528 (Diseases of the nervous system - Disorders of other specified cranial nerves)	N	P
S392	H814 (Diseases of the ear and mastoid - Vertigo of central origin)	N	P
S393	G459 (Diseases of the nervous system - Transient cerebral ischemic attacks and related syndromes)	P	N
S394	G439 (Diseases of the nervous system - Migraine, unspecified)	N	N
S395	G459 (Diseases of the nervous system - Transient cerebral ischemic attacks and related syndromes)	N	N
S396	R51 (Symptoms, signs, and clinical and laboratory abnormalities not otherwise specified - Headache)	N	N
S397	H812 (Diseases of the ear and mastoid - Vestibular neuronitis)	N	N
S398	H532 (Diseases of the eye and eye appendages - Double vision)	N	N
S399	H814 (Diseases of the ear and mastoid - Vertigo of central origin)	N	N
S400	M5459 (Musculoskeletal system and connective tissue diseases - Low back pain, site unspecified)	N	P
S401	G459 (Diseases of the nervous system - Transient cerebral ischemic attacks and related syndromes)	N	N
S402	T424 (Injury, poisoning, and other specific consequences of exogenous factors - Benzodiazepine)	N	P
S403	G459 (Diseases of the nervous system - Transient cerebral ischemic attacks and related syndromes)	N	N
S404	G459 (Diseases of the nervous system - Transient cerebral ischemic attacks and related syndromes)	N	P
S405	R471 (Symptoms, signs, and clinical and laboratory abnormalities not otherwise specified - Dysarthria and anarthria)	P	N
S406	G454 (Diseases of the nervous system - Transient global amnesia)	N	N
S407	G459 (Diseases of the nervous system - Transient cerebral ischemic attacks and related syndromes)	N	N
S408	G454 (Diseases of the nervous system - Transient global amnesia)	N	P
S409	G459 (Diseases of the nervous system - Transient cerebral ischemic attacks and related syndromes)	N	P
S410	H814 (Diseases of the ear and mastoid - Vertigo of central origin)	N	N
S411	G4090 (Diseases of the nervous system - Epilepsy, unspecified without intractable epilepsy)	N	N
S412	G454 (Diseases of the nervous system - Transient global amnesia)	N	N
S413	G459 (Diseases of the nervous system - Transient cerebral ischemic attacks and related syndromes)	N	N
S414	R42 (Symptoms, signs, and clinical and laboratory abnormalities not otherwise specified - Dizziness)	N	N

S415	G459 (Diseases of the nervous system - Transient cerebral ischemic attacks and related syndromes)	N	N
S416	G459 (Diseases of the nervous system - Transient cerebral ischemic attacks and related syndromes)	N	N
S417	R550 (Symptoms, signs, and clinical and laboratory abnormalities not otherwise specified - Vasovagal syncope)	N	N
S418	H814 (Diseases of the ear and mastoid - Vertigo of central origin)	P	P
S419	R51 (Symptoms, signs, and clinical and laboratory abnormalities not otherwise specified - Headache)	N	N
S420	H814 (Diseases of the ear and mastoid - Vertigo of central origin)	P	N
S421	G459 (Diseases of the nervous system - Transient cerebral ischemic attacks and related syndromes)	P	N
S422	G459 (Diseases of the nervous system - Transient cerebral ischemic attacks and related syndromes)	N	N
S423	H814 (Diseases of the ear and mastoid - Vertigo of central origin)	N	N
S424	H814 (Diseases of the ear and mastoid - Vertigo of central origin)	N	N
S425	G459 (Diseases of the nervous system - Transient cerebral ischemic attacks and related syndromes)	N	P
S426	G459 (Diseases of the nervous system - Transient cerebral ischemic attacks and related syndromes)	N	N
S427	H814 (Diseases of the ear and mastoid - Vertigo of central origin)	N	N
S428	R42 (Symptoms, signs, and clinical and laboratory abnormalities not otherwise specified - Dizziness)	N	N
S429	G459 (Diseases of the nervous system - Transient cerebral ischemic attacks and related syndromes)	N	P
S430	G459 (Diseases of the nervous system - Transient cerebral ischemic attacks and related syndromes)	N	N
S431	G459 (Diseases of the nervous system - Transient cerebral ischemic attacks and related syndromes)	N	N
S432	Z027 (Factors affecting health status and access to health services - Issue of medical certificate)	N	N
S433	G459 (Diseases of the nervous system - Transient cerebral ischemic attacks and related syndromes)	P	P
S434	H819 (Diseases of the ear and mastoid - Disorder of vestibular function, unspecified)	N	P
S435	M512 (Musculoskeletal system and connective tissue diseases - Other specified intervertebral disc displacement)	N	N
S436	R558 (Symptoms, signs, and clinical and laboratory abnormalities not otherwise specified - Other syncope and collapse)	P	N
S437	H814 (Diseases of the ear and mastoid - Vertigo of central origin)	N	N
S438	H814 (Diseases of the ear and mastoid - Vertigo of central origin)	N	N
S439	G454 (Diseases of the nervous system - Transient global amnesia)	N	N
S440	G459 (Diseases of the nervous system - Transient cerebral ischemic attacks and related syndromes)	N	P
S441	H814 (Diseases of the ear and mastoid - Vertigo of central origin)	N	N

S442	R568 (Symptoms, signs, and clinical and laboratory abnormalities not otherwise specified - Other and unspecified convulsions)	N	N
S443	S134 (Injury, poisoning, and other specific consequences of exogenous factors - Sprain and strain of cervical spine)	N	N
S444	G459 (Diseases of the nervous system - Transient cerebral ischemic attacks and related syndromes)	P	N
S445	E512 (Endocrine, nutritional and metabolic diseases - Wernicke's encephalopathy)	P	N
S446	H814 (Diseases of the ear and mastoid - Vertigo of central origin)	N	P
S447	R558 (Symptoms, signs, and clinical and laboratory abnormalities not otherwise specified - Other syncope and collapse)	N	N
S448	G459 (Diseases of the nervous system - Transient cerebral ischemic attacks and related syndromes)	N	N
S449	H814 (Diseases of the ear and mastoid - Vertigo of central origin)	N	N
S451	G459 (Diseases of the nervous system - Transient cerebral ischemic attacks and related syndromes)	P	N
S452	G839 (Diseases of the nervous system - Paralytic syndrome)	N	N
S453	G439 (Diseases of the nervous system - Migraine, unspecified)	N	P
S454	G562 (Diseases of the nervous system - Lesion of ulnar nerve)	N	P
S455	M7929 (Musculoskeletal system and connective tissue diseases - Neuralgia and neuritis, unspecified, site unspecified)	N	N
S456	G459 (Diseases of the nervous system - Transient cerebral ischemic attacks and related syndromes)	P	N
S457	G459 (Diseases of the nervous system - Transient cerebral ischemic attacks and related syndromes)	N	P
S458	G459 (Diseases of the nervous system - Transient cerebral ischemic attacks and related syndromes)	N	P
S459	H8138 (Diseases of the ear and mastoid - Other peripheral vertigo)	P	N
S460	R51 (Symptoms, signs, and clinical and laboratory abnormalities not otherwise specified - Headache)	N	N
S461	R568 (Symptoms, signs, and clinical and laboratory abnormalities not otherwise specified - Other and unspecified convulsions)	N	P
S462	G459 (Diseases of the nervous system - Transient cerebral ischemic attacks and related syndromes)	N	N
S463	F449 (Mental and behavioral disorders - Dissociative disorder, unspecified)	N	N
S464	G439 (Diseases of the nervous system - Migraine, unspecified)	N	N
S465	G459 (Diseases of the nervous system - Transient cerebral ischemic attacks and related syndromes)	N	P
S466	H814 (Diseases of the ear and mastoid - Vertigo of central origin)	N	P
S467	G459 (Diseases of the nervous system - Transient cerebral ischemic attacks and related syndromes)	N	N
S468	R42 (Symptoms, signs, and clinical and laboratory abnormalities not otherwise specified - Dizziness)	N	N

S469	R558 (Symptoms, signs, and clinical and laboratory abnormalities not otherwise specified - Other syncope and collapse)	N	N
S470	R509 (Symptoms, signs, and clinical and laboratory abnormalities not otherwise specified - Fever, unspecified)	N	N
S471	H814 (Diseases of the ear and mastoid - Vertigo of central origin)	N	N
S472	G459 (Diseases of the nervous system - Transient cerebral ischemic attacks and related syndromes)	P	N
S473	R51 (Symptoms, signs, and clinical and laboratory abnormalities not otherwise specified - Headache)	N	N
S474	G459 (Diseases of the nervous system - Transient cerebral ischemic attacks and related syndromes)	N	P
S475	G459 (Diseases of the nervous system - Transient cerebral ischemic attacks and related syndromes)	N	N
S476	G459 (Diseases of the nervous system - Transient cerebral ischemic attacks and related syndromes)	N	N
S477	R42 (Symptoms, signs, and clinical and laboratory abnormalities not otherwise specified - Dizziness)	N	N
S478	R558 (Symptoms, signs, and clinical and laboratory abnormalities not otherwise specified - Other syncope and collapse)	N	P
S479	R42 (Symptoms, signs, and clinical and laboratory abnormalities not otherwise specified - Dizziness)	N	P
S480	G459 (Diseases of the nervous system - Transient cerebral ischemic attacks and related syndromes)	P	N
S481	G459 (Diseases of the nervous system - Transient cerebral ischemic attacks and related syndromes)	N	N
S482	R558 (Symptoms, signs, and clinical and laboratory abnormalities not otherwise specified - Other syncope and collapse)	N	N
S483	H814 (Diseases of the ear and mastoid - Vertigo of central origin)	N	N
